# Experimental and Theoretical Analysis of Dopamine Polymerization on the Surface of Cellulose Nanocrystals and Its Reinforcing Properties in Cellulose Acetate Films

**DOI:** 10.3390/polym17030345

**Published:** 2025-01-27

**Authors:** Amanda Lélis de Souza, Arthur Vinicius de Abreu Oliveira, Laisse Dias Ribeiro, Allan Robledo Fialho e Moraes, Meirielly Jesus, Joana Santos, Taila Veloso de Oliveira, Nilda de Fátima Ferreira Soares

**Affiliations:** 1 Laboratory of Polymeric Materials, Food Technology Department, Federal University of Viçosa, Viçosa CEP 36570-000, Minas Gerais, Brazil; amandalelis247@gmail.com (A.L.d.S.); taila.oliveira@ufv.br (T.V.d.O.); 2 Laboratory of Biochemical and Fermentative Processes, Food Technology Department, Federal University of Viçosa, Viçosa CEP 36570-000, Minas Gerais, Brazil; arthur.abreu@ufv.br; 3 Institute of Science and Technology, Federal University of Jequitinhonha and Mucuri Valleys, Diamantina CEP 39100-000, Minas Gerais, Brazil; laisse.ribeiro@ict.ufvjm.edu.br; 4 Institute of Agricultural Sciences, Federal University of Viçosa, Rio Paranaíba CEP 38810-000, Minas Gerais, Brazil; allan.moraes@ufv.br; 5 CISAS-Center for Research and Development in Agrifood Systems and Sustainability, Polytechnic Institute of Viana do Castelo, Rua da Escola Industrial e Comercial Nun’Alvares 34, 4900-347 Viana do Castelo, Portugal; joana@estg.ipvc.pt

**Keywords:** biomimetics, polydopamine, hydrophobic polymer

## Abstract

The study of natural materials inspires sustainable innovations, with biomimetics excelling in surface modification. Polydopamine (PDA) offers a promising approach for modifying cellulose nanocrystals (CNC), enhancing their compatibility with hydrophobic polymers by improving interfacial adhesion. In this work, the modification of CNC with PDA (CNC@PDA) significantly enhanced the compatibility between the nanocargoes and the cellulose acetate (CA) matrix. The CNC@PDA complex formation was suggested through a combination of FTIR analysis, particle size distribution measurements and *ζ*-potential analysis. However, the exact mechanism behind dopamine polymerization on the surface of CNC remains a subject of ongoing debate among researchers due to its complexity. This study hypothesized the formation of modified CNC through this process. Furthermore, this study provided a satisfactory investigation of the antimicrobial activity of CNC@PDA in response to bacterial strains (*E. coli*, *P. aeruginosa*, *S. aureus* and *L. plantarum*) in view of the hypothesis of the possible generation of reactive oxygen species (ROS). Additionally, the incorporation of CNC@PDA CA films was analyzed to assess its effect as a mechanical reinforcement agent. The results showed an improvement in mechanical properties, with the 1% CNC@PDA film exhibiting the best balance between tensile strength and flexibility.

## 1. Introduction

Cellulose nanocrystals (CNCs) stand out as one of the most widely used nanomaterials, mainly due to their high surface-to-volume ratio and the presence of hydroxyl groups that allow for convenient functionalization [1]. Not only do these nanocrystals exhibit a remarkable tensile strength (10 GPa) and a significant stiffness (110–130) GPa [2], but they are also derived from cellulose, the most abundant renewable natural polymer, composed of β-1,4-d-glucose units [3]. This abundance and its properties make cellulose an essential material in industry, playing a crucial role in sustainable development [4].

Despite its remarkable qualities, CNC has some limitations, such as non-uniform behavior and low adhesiveness in polymeric matrices [5,6]. To address these shortcomings, strategies for modifying the surface of CNC at the nanoscale have been developed. One promising approach is the functionalization of the CNC surface, which allows the creation of biocompatible and biodegradable nanostructures with customized physical and chemical properties. Recently, dopamine polymerization, polydopamine (PDA), has proved to be an effective technique in this context [7,8].

PDA is a biomimetic polymer obtained by the oxidative self-polymerization of dopamine under alkaline and humid conditions [9]; it is inspired by the Mefp-5 protein from marine mussels, known for its strong adhesion to various materials [10]. PDA’s ability to deposit quickly and stably on almost all inorganic and organic substrates gives it a significant advantage [11]. Due to its characteristic adhesiveness, optical properties, biocompatibility and low cytotoxicity, PDA has been widely used as a substrate modifier for the construction of multifunctional materials [12].

Several studies have investigated the modification of CNC with PDA. For example, CNC was modified with PDA as nanocarriers for the modification of poly lactic acid (PLA) [12]. Another study focused on the self-polymerization of dopamine to coat CNC surfaces, integrating functionalities and structural merits into polyvinyl alcohol (PVA) films [8]. In addition, CNC was grafted with polyethylene glycol (PEG) using PDA as a secondary reaction platform to obtain CNC-PEG, which was subsequently used to reinforce PVA films [13].

Although these studies indicate that the introduction of PDA is an effective strategy for modifying the surface of CNC and building multifunctional composites, the exact interaction between PDA and CNC has not yet been fully elucidated. Therefore, this work aimed to modify the surface of CNC through dopamine polymerization, characterize the resulting physicochemical changes, and evaluate the improvement in compatibility with hydrophobic polymer matrices. Additionally, the incorporation of PDA-coated CNC (CNC@PDA) into cellulose acetate (CA) films was investigated, analyzing its role as a mechanical reinforcement agent, with the aim of developing materials with enhanced structural performance.

## 2. Materials and Methods

### 2.1. Materials

The materials included cellulose nanocrystals (CelluForce, Pointe-Claire, QC, Canada), dopamine hydrochloride (Sigma-Aldrich, St. Louis, MO, USA), tris(hydroxymethyl)aminomethane (Tris) (Sigma-Aldrich, St. Louis, USA), ultrapure water (electrical resistivity of 18.2 MΩ·cm (Millipore Inc., Burlington, NJ, USA), Mueller Hinton broth (Kasvi, São Paulo, Brazil), Mueller Hinton agar (Becton Dickinson, Franklin Lakes, NJ, USA), cellulose acetate (donated by Rodhia, São Paulo, Brazil) and triethyl citrate (Sigma-Aldrich, St. Louis, MO, USA). All the chemical reagents used were of analytical grade.

### 2.2. Preparation of Cellulose Nanocrystals (CNCs) Coated with Polydopamine (CNC@PDA)

The surface modification of the CNCs was carried out by dopamine polymerization according to the method of Nagamine et al. (2021), with modifications [14]. Initially, 1 g of CNC was dispersed in 100 mL of ultrapure water under magnetic stirring for 12 h at room temperature (25 ± 2 °C) to ensure uniform dispersion and removal of soluble impurities. The suspension was adjusted to pH 8.5 ± 0.1 using Tris-HCl buffer solution (10 mM, pH 8.5) to create optimal conditions for dopamine polymerization while neutralizing potential contaminants. Then, dopamine hydrochloride (0.05 g·L^−1^) was added under magnetic stirring for 24 h at 25 ± 2 °C to proceed with the formation of PDA-coated CNC (CNC@PDA). The process effectively reduced impurities through controlled reaction conditions and buffer utilization, ensuring the successful coating of CNC with PDA.

For the scanning electron microscopy (SEM), Fourier-transformed infrared absorption spectroscopy (FTIR) and thermogravimetric analysis (TGA), 50 g of the CNC@PDA suspensions were dried in a freeze-dryer (Christ, model Alpha 2-4 LD plus) at −40 °C and 0.12 mbar for 48 h.

### 2.3. Characterization of CNC@PDA

An amount of 2 g of each previously homogenized suspension was weighed, then dried in a forced circulation oven (Tecnal Equipamentos Científicos Ltd., São Paulo, Brazil) at 105 °C for 24 h. The dried samples were characterized as follow.

The ζ potential was estimated for suspensions of CNC, PDA and CNC@PDA. Aliquots of 1 mL of the suspensions were placed in a cuvette (disposable folded capillary cell), placed inside the equipment and exposed to a constant electric field (fixed angle of 173°) at a temperature of 25 ± 2 °C (Malvern Panalytical, Grovewood, UK). The speed and direction of the particle’s movement due to the electric field allowed the electrophoretic mobility to be calculated (Equation (1)). Next, the Smoluchowski model (Equation (2)) for the electric double layer was considered to calculate the values *ζ* potential.

(1) $$\mu_{e}=\frac{v}{\overset{\to }{E}}$$

(2) $$\zeta =\frac{\eta \mu_{e}}{\varepsilon_{0}\varepsilon_{r}}$$

In Equations (1) and (2), $\mu_{e}$ is the electrophoretic mobility, v is the particle velocity, $\overset{\to }{E}$ is the electric field, $\varepsilon_{r}$ is the dielectric constant of the medium, $\varepsilon_{0}$ is the permittivity of free space, ζ is the zeta potential and η is the viscosity of the medium.

The mean hydrodynamic diameter $d_{h}$ and particle distribution index (PDI) were evaluated by dynamic light scattering (DLS) (Ζsizer NanoZS, Malvern Instruments, UK). For this analysis, the colloidal dispersions were diluted (1:10) in ultrapure water. Each system to be analyzed was then placed in a cuvette and the analyses were all carried out at 25 ± 2 °C.

The hydrodynamic diameter distributions were obtained by means of the amplitude of the decay rate A(Γ), obtained by adjusting the normalized time intensity correlation functions, $g^{\left(2\right)}\left(t\right)$, by means of an NNLS (Non-Negative Least Square) algorithm, according to Equation (3) [15]. Then, the distribution *Γ* was transformed into $d_{h}$ sequentially using Equations (4) and (5):

(3) $$g^{\left(2\right)}\left(t\right)=1+\beta {\left[\int_{0}^{\infty }A(\Gamma )e^{-\Gamma t}dt\right]}^{2}$$

(4) $$\Gamma =D{\left(\frac{4\pi \eta_{i}}{\lambda }\sin\frac{\theta }{2}\right)}^{2}$$

(5) $$D=\frac{k_{B}T}{3\pi \eta d_{h}}$$

In Equations (3)–(5), *A* and β are constants that depend on the number of coherence areas in the detector, Γ is the decay rate, $\eta_{i}$ is the refractive index of the dispersion, λ is the laser wavelength, θ is the detection angle, D is the mass diffusivity of the dispersed particles, $k_{B}$ is Boltzmann’s constant, T is the absolute temperature, η is the viscosity of the medium and *d_h_* is the average hydrodynamic diameter of the dispersed particles. The polydispersity index (PDI) was calculated for each size distribution estimated according to Equation (6) [16]:

(6) $$PDI={\left(\frac{SD}{d_{h}}\right)}^{2}$$

In Equation (6), SD is the standard deviation corresponding to each $d_{h}$ value.

FTIR spectroscopy was used to examine the molecular interactions between PDA and CNC, such as electrostatic interactions and hydrogen bonds. The spectra were carried out using a spectrophotometer Cary 630 FTIR (Agilent Technologies, Santa Clara, CA, USA) equipped with an attenuated reflectance (ATR) accessory in the infrared region from 4000 to 650 cm^−1^, with 32 scans and 4 cm^−1^ spectral resolution. This analysis was conducted to observe the changes in functional groups before and after the interactions between PDA and CNC as an indication of the formation of the graft (CNC@PDA).

The thermal behavior of the samples was investigated using a thermogravimetric analyzer (DTG-60H, Shimadzu, Kyoto, Japan) in a dynamic nitrogen atmosphere (50 mL^·^min^−1^) with a heating rate of 10 °C·min^−1^, in a temperature range between 25 °C and 600 °C. For this analysis, approximately 3 mg of each sample packed in alumina crucibles were weighed on a TGA microbalance (Mettler Toledo, Columbus, OH, USA).

### 2.4. Investigation of the Antimicrobial Activity of Polydopamine Solution and CNC@PDA Suspension

The antimicrobial activity of the PDA solution and CNC@PDA suspension were investigated against the following microorganisms: *Escherichia coli* ATCC 11229, *Pseudomonas aeruginosa* ATCC 6538, *Staphylococcus aureus* ATCC 15442 and *Lactobacillus Plantarum* ATCC 8014. The microorganism inocula, previously stored at −80 ± 2 °C in the Laboratory of Polymeric Materials ultrafreezer, were reactivated twice in Mueller Hinton broth (37 ± 2 °C, 24 h). Suspensions of turbidity equivalent to the McFarland 0.5 standard (approximately 1 × 10^8^ CFU·mL^−1^) were then prepared from 0.85% (*w*/*v*) saline solution.

#### Agar Diffusion Plate Method

The agar diffusion plate method was used to evaluate the antimicrobial activity of the PDA solution and the CNC@PDA suspension according to the recommendations of the Clinical and Laboratory Standards Institute (2012), with modifications [17]. The inoculums were prepared as described above. Approximately 15 mL of Mueller Hinton agar at 45 ± 2 °C was poured into sterilized petri dishes and solidified at 25 ± 2 °C under aseptic conditions. For the PDA solution and CNC@PDA suspension, 6 mm diameter holes were drilled in the solid agar. Using a swab, the inoculum was applied to the surface of the dry medium, and then 5 μL aliquots of the dopamine solution or CNC@PDA suspension were added to the holes. The plates were incubated at 35 ± 2 °C for 24 h. The inhibition halos formed were measured.

### 2.5. Production and Mechanical Characterization of Cellulose Acetate-Based Films Using CNC@PDA

The cellulose acetate (CA) films were produced using the casting method, according to the methodology described by Soares and Hotchkiss (1998) with modifications [18]. First, 3 g of CA was dispersed in 30 mL of acetone, which was used as the solvent. Subsequently, 15% (m/m) triethyl citrate was added to this solution as a plasticizer. This dispersion was sealed with parafilm and left to stand for 24 h to allow the CA to completely disperse in the liquid phase of the polymer dispersion.

After 24 h, CNC@PDA was inserted into the polymer dispersion at different concentrations of 0, 0.5, 1, 1.5 and 2% (*m*/*v*) and stirred with the aid of an ultra-turrax at 20,000 rpm for 1 min, then left to rest for 10 min and spread onto glass plates with the aid of an applicator (K Paint applicator). After the solvent had evaporated under ambient conditions (25 ± 2 °C), the films were removed from the plates and packed in polyethylene/nylon containers until use.

#### Mechanical Properties Analysis

The films produced were subjected to mechanical tests to assess tensile strength (MPa) and elongation at break (%) according to standard method D882-12 (ASTM, 2012) using the Universal Mechanical Testing Machine 3367 (Instron Corporation, Norwood, MA, USA). Five specimens of each treatment were evaluated, with dimensions of 175 mm in length and 25 mm in width. The machine was operated with a load of 1 kN, a claw separation speed of 50 mm·min^−1^ and an initial distance of 125 mm. Tensile strength is the maximum load divided by the cross-sectional area of the film, and elongation at break represents the percentage ratio between the elongation of the specimen before breaking and its initial length.

### 2.6. Statistical Analysis

To compare the differences between the mean properties of CNC, PDA and CNC@PDA and the CNC@PDA-CA films, analysis of variance (ANOVA) and Tukey’s multiple comparisons test with a 10% significance level were used with the aid of Statistica 7.0 software (StatSoft Inc., Tulsa, OK, USA).

## 3. Results and Discussion

### 3.1. Preparation and Characterization of CNC@PDA Suspensions

**Figure 1:**
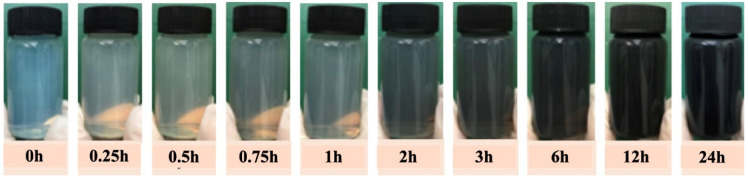
Color change as an indication of dopamine polymerization on the surface of cellulose nanocrystals between 0 and 24 h.

Under aerobic, alkaline conditions and at room temperature (25 ± 2 °C), the polymerization of dopamine on the surface of the CNC transformed the initially clear suspension into an aqueous suspension of modified CNC (CNC@PDA) with a dark (black) color. This dark coloration is a typical characteristic of polydopamine coatings [12,14]. Figure 1 illustrates the change in coloration over time from 0 to 24 h, visually indicating the progress and success of dopamine polymerization on the surface of the CNC. The aqueous suspension of the CNC@PDA resulted in an average concentration of 0.70 g of CNC@PDA/100 g of suspension.

The color change in the CNC@PDA suspension occurs due to two interconnected processes. First, the polymerization of dopamine results in the formation of PDA. During this process, the aromatic units (benzene rings) interact electrically, causing electron delocalization within the structure, which gives PDA its dark color. Second, the interaction of dopamine with the CNC surface, through hydrogen bonds and π-π interactions, stabilizes the formed polymer layer, ensuring strong adhesion and maintaining the dark color due to the structural uniformity of PDA.

**Figure 2:**
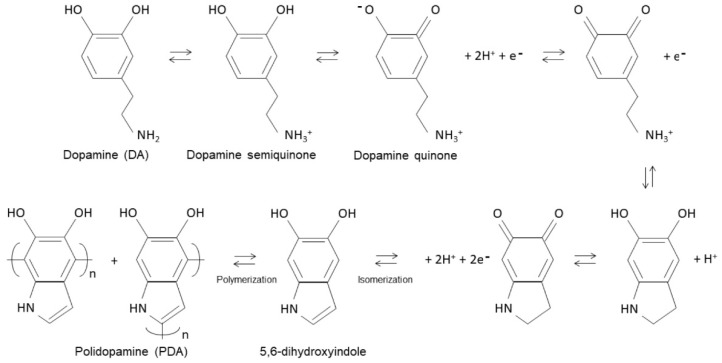
Schematic of the mechanism of dopamine polymerization.

The efficiency of this modification is influenced by various factors, with the pH of the aqueous medium being one of the most critical. The pH affects the proportion of amino and phenolic groups involved in the polymerization of dopamine, an essential process for the formation of PDA. Although the fundamental mechanism for the formation of PDA is still not fully understood [19], the polymerization of dopamine generally follows the chemical process described by Lee [20]. Figure 2 illustrates a possible schematic of the polymerization mechanism, showing that the molecular structure of dopamine has two phenolic groups that can be deprotonated and a primary amino group that can be protonated, respectively.

This article explores the chemical process described by Lee [20] in more depth. When the pH of a solution is lower than 8.5, say around 7.5, dopamine, which is a molecule containing primary amines and hydroxyl groups, behaves as follows:

(1) Protonated primary amine: At this lower pH, dopamine’s primary amine is protonated. This means that it has captured a hydrogen ion (H⁺) from the solution, becoming positively charged (dopamine semiquinone). This is because, in acidic or slightly neutral environments, amines generally accept protons and become protonated. In other words, the protonated form is dominant because the pH is below the pKa of the primary amine.
(2) Undissociated hydroxyl groups: While the amine is protonated, the hydroxyl groups (–OH) in dopamine are not dissociated. Dissociation, in this context, refers to the separation of a hydrogen from the hydroxyl group, forming phenoxide ions (–O^−^). At a pH below 8.5, these hydroxyl groups remain in their original form and do not dissociate (dopamine semiquinone).
(3) Transition ≈ pH 8.5: As the pH of the solution increases and approaches 8.5, the environment becomes more alkaline. At this higher pH, the hydroxyl group of dopamine, which is more acidic than the amine, begins to dissociate (dopamine quinone). This means that the hydroxyl group can lose a hydrogen ion, forming phenoxide ions (–O^−^) in the solution.
(4) Equilibrium: When the pH is close to 8.5, an equilibrium is established between the protonated form of the amine and the dissociation of the hydroxyl group. This means that, from this point onwards, both the protonated form of the amine and the dissociated hydroxyl groups coexist in a proportion determined by the pH of the solution.

The proposed mechanism for oxidation at pH = 8.5 is explained by an electron transfer reaction that creates a semiquinone radical. This radical is an intermediate in the reaction and is generated by an electron transfer. The semiquinone radical can be further oxidized to form dopamine quinone. Dopamine quinone contains an electron-deficient ring and an electron-donating amine group. The dopamine quinone can undergo a 1,4 Michael addition with another dopamine molecule or an intermediate, and the amine group can be deprotonated, leading to an intramolecular cyclization reaction. The oxidation of dopamine quinone and the subsequent cyclization reaction can form 5,6-dihydroxyindol. This compound is an important precursor in the polymerization of dopamine, as it is capable of self-polymerizing, resulting in the formation of PDA. The pH value used in this research to induce the self-polymerization of dopamine was 8.5 (Tris-HCl 10 mM) in order to keep the pH constant to ensure the efficiency and stability of the reaction.

The exact adhesive mechanism (synthesis) is still not widely known; however, it is widely accepted that PDA acts as a universal biocola, capable of adhering to a variety of surfaces, both organic and inorganic [21,22,23]. With this in mind, some hypotheses can be hypothesized that influence the formation of CNC@PDA: (i) the PDA may adhere to the CNC through hydrogen bonds with the hydroxyl groups of the CNC, facilitating adhesion and favoring conformational changes in the CNC structure; (ii) interaction between the functional groups on the PDA and the carbon atoms on the surface of the CNC may form coordination bonds that contribute to adhesion; (iii) additional association between CNC and PDA, influenced by the attraction between local clusters in supramolecular structures, can be affected by steric hindrances caused by bulky groups, and side chains (both PDA and CNC) can make the structure less ordered and affect the interaction; (iv) PDA can form hydrophobic interactions with hydrophobic or semi-hydrophobic regions on the surface of CNC, as PDA has regions in its structure that can be less polar and therefore hydrophobic. These regions can interact with areas of the CNC surface that also have hydrophobic characteristics, even though the CNC surface is predominantly hydrophilic due to the hydroxyl groups. (v) Intermolecular hydrogen bonds can form. PDA can form hydrogen bridges with other functional groups present on the surface of CNC or between different PDA molecules. (vi) Additional chemical reactions, such as oxidation or cross-linking reactions, can occur between dopamine or PDA and the groups on the surface of CNC. PDA can react chemically with the functional groups on the surface of the CNC, as well as interacting physically, affecting the formation of the coating. (vii) The polymerization of dopamine can cause changes in the structure and properties of the CNC, influencing how the PDA adheres. During polymerization, dopamine can form complex networks or structures that affect the interaction with CNC.

### 3.2. ζ Potential

The CNC@PDA were characterized in terms of ζ potential, average hydrodynamic diameter and polydispersity index (Table 1).

The ζ potential of the CNC changed from −67.53 ± 0.87 mV to −39.0 ± 1.36 mV after modification with PDA, a result similar to that reported by [23]. The reduction in charge density (modulus) suggests a decrease in the overall negative charge (anionic structure) of the unmodified CNC, which is expected due to the addition of PDA. The decrease in charge density can be explained by the interaction of PDA containing catechol and amine functional groups with the negatively charged sulfate ester groups on the CNC surface, effectively neutralizing part of the original negative charge. In addition to the total charge density, other factors contribute to a more complete understanding of the surface modification and the interactions involved, such as the local charge distribution on the polysaccharide chains, the position of the charged groups, the steric factors and the flexibility of the chains, which also affect how PDA interacts with the surface of the CNC [24].

All the studied mixtures exhibited a monomodal size distribution, with peaks below 200 nm. The hydrodynamic diameter of the CNC was initially 85.95 ± 2.19 nm, and after modification, it increased to 274.93 ± 3.6 nm, indicating an increase in the average particle diameter. It is important to note that the hydrodynamic diameter is a measure that describes the effective size of a particle in suspension based on its behavior in a fluid and not just its direct physical size. In the case of CNC, this diameter takes into account the particle’s interaction with the dispersing medium, including the effects of molecule adsorption around the particle and the influence of electrostatic forces [25]. An increase in hydrodynamic diameter is associated with a low ionic concentration, resulting in an extended ion layer around the particle, which decreases its diffusion rate. This effect is consistent with the zeta potential results, which show a reduction in charge density (modulus), indicating an interaction between the CNC and the PDA. In contrast, a high ionic concentration compresses the ion layer, resulting in a smaller hydrodynamic diameter for the same particle.

Although the hydrodynamic diameter of CNC@PDA was similar to that of pure PDA, it is possible that the adsorption of PDA onto CNC did not result in a significant increase in the average particle diameter due to the type of interaction between the two materials. The adsorption may be superficial or occur in a thin layer around the CNC, not substantially altering the size of the particle in suspension.

The polydispersity index (PDI) is an essential parameter that describes the breadth of the particle size distribution [26]. The PDI provides an indication of how particle sizes are distributed in a sample; lower values indicate a narrower size distribution, while higher values reflect a broader and more varied distribution. After the modification of CNC to CNC@PDA, a high PDI value (0.81) was observed. This result suggests that after modification, the particles have a broader size distribution, which may be due to various interactions between the particles and/or between the particles and the dispersing medium. These interactions can lead to the formation of aggregates or variations in particle size within the sample due to the amount of PDA incorporated and the presence of pristine particles.

### 3.3. Fourier-Transformed Infrared Spectroscopy (FTIR)

**Figure 3:**
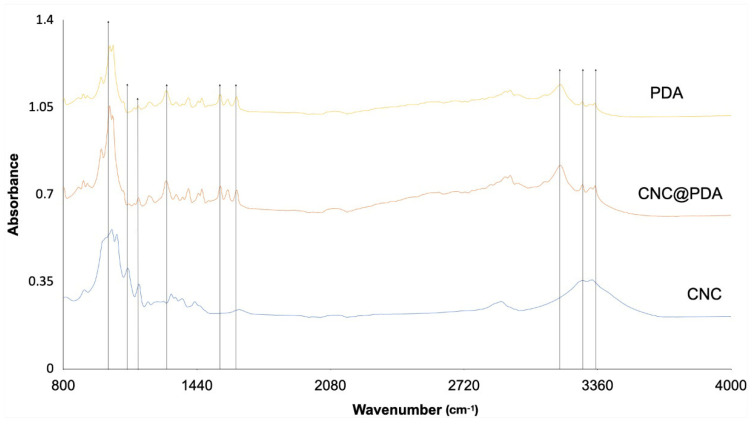
FTIR spectra of CNC, PDA and CNC@PDA.

FTIR technique was used to analyze the functional groups present in the samples. Figure 3 shows the spectra of CNC, PDA and CNC@PDA.

The broad absorption range in the region of 3650 to 3000 cm^−1^ is attributed to the hydroxyl group (–OH). This group is characteristic of the hydroxyl groups present in both CNC and PDA, reflecting the hydrophilic tendency of the samples. Theoretically, the absorption in this range occurs due to O–H stretching vibrations, which indicate the sample’s ability to form hydrogen bonds and thus interact favorably with water.

The shift in wavenumber observed in FT-IR occurs due to changes in the strength or geometry of chemical bonds. In CNC@PDA, the peak at 3200 cm^−1^ tends to intensify and, in some cases, slightly shift, indicating the formation of new hydrogen bond interactions between the functional groups of CNC and PDA. This intensification, accompanied by band broadening, reflects the increase in –OH and –NH groups on the CNC surface, characterizing functionalization.

However, the absence of significant shifts in wavenumber between the spectra of CNC@PDA and PDA can be explained. The interactions formed, such as OH...NH or OH...OH, may be similar to those already present in PDA, masking more evident changes. Additionally, the –OH and –NH functional groups exhibit broad and overlapping bands, which makes subtle shifts difficult to detect [12,23].

Another factor is that PDA exhibits dominant bands associated with its functional groups, such as catechols, amines and quinones, which remain similar after functionalization. This can cause the overall spectrum of CNC@PDA to resemble that of PDA, even with the intensification of specific bands. Very small shifts, on the order of a few cm^−1^, may also go unnoticed due to the limitations of the technique. Nonetheless, the intensification and broadening of the band at 3200 cm^−1^ provide strong evidence that the functional groups of PDA are present on the CNC surface.

The peak at 1630 cm^−1^ is attributed to the stretching vibrations of C=C bonds in the aromatic rings of PDA, which are formed during dopamine polymerization. Since CNC does not contain aromatic rings in its structure, the absence of this peak in the FTIR spectrum of CNC reinforces that the observed peak in CNC@PDA is indeed related to PDA and not to components of CNC. This result suggests that PDA has been adsorbed onto the surface of CNC, bringing its aromatic groups with it, but without necessarily interacting chemically in a strong way with CNC, as the PDA peak remains unchanged.

Therefore, the presence of the peak at 1630 cm^−1^ in CNC@PDA suggests the incorporation of PDA onto the surface of CNC but does not necessarily imply complex chemical interactions between the two materials, indicating that it is likely only a physical coating.

The peak at 1560 cm^−1^, observed in the spectra of CNC@PDA and PDA but absent in CNC, is associated with the stretching vibrations of C=C in aromatic rings and the bending vibrations of N–H [11,12]. The absence of a shift in the wavenumber, despite the presence of the peak at 1580 cm^−1^ in CNC@PDA, suggests that PDA was adsorbed onto the surface of the CNC without significantly altering the characteristics of the bonds in the PDA. This implies that the modification of CNC with PDA likely involved physical interactions (such as π-π interactions and weak hydrogen bonding), rather than strong chemical bonds. The intensification of the peak can be explained by the increased amount of PDA functional groups on the surface of the CNC, but the lack of a wavenumber shift indicates that these interactions were not strong enough to alter the vibrations of the C=C or N-H bonds, typical of the aromatic rings and amino groups in PDA. Therefore, we cannot assert that new complex chemical interactions occurred, but rather that the PDA groups were physically incorporated onto the surface of the CNC.

The peak at 1285 cm^−1^ observed in CNC@PDA and PDA is associated with the bending vibrations of phenolic O–H bonds [11,12]. This peak is characteristic of the phenolic groups in PDA, which are responsible for hydrogen bonding and phenolic interactions within the material structure. The presence of this peak in CNC@PDA does not necessarily indicate new interactions or modifications in the phenolic bonds, as this peak is already present in pure PDA. Instead, it suggests that the phenolic groups of PDA have been retained on the surface of the CNC during the modification process. The intensity of the peak may reflect the amount of phenolic groups present on the CNC surface, but without implying complex chemical interactions between PDA and CNC. Therefore, the presence of this peak is an indication that PDA has been incorporated into the CNC surface without significant alteration of the structural characteristics of the phenolic groups.

The peak at 1200 cm^−1^ observed in CNC@PDA and PDA is associated with C–O stretching, which is characteristic of phenolic groups and ethers in the structure of PDA [11,12]. This peak is evidence of the presence of functional groups containing C–O bonds, which are common in the phenolic structure of PDA. When present in CNC@PDA, this peak indicates that the phenolic groups of PDA were effectively incorporated onto the surface of the CNC.

The peak at 1110 cm^−1^ is prominent and indicates the significant presence of C–O bonds, reflecting the polysaccharide structure of CNC [11,12]. In CNC@PDA, a change in intensity or even disappearance of this peak suggests that PDA is covering the surface of the CNC and altering how the C–O vibrations from the CNC are recorded in the FTIR spectrum. Moreover, the dilution of C–O groups from CNC in the CNC@PDA composition may also contribute to this alteration. In PDA, this peak may be absent or very weak, as PDA does not contain the same C–O groups found in CNC.

This change in the spectrum does not indicate a direct chemical reaction between PDA and C–O groups, but rather a modification of the CNC surface due to PDA adsorption. When PDA is adsorbed, it may form a layer over the CNC, altering how the C–O groups interact with infrared radiation. This can occur due to physical interactions between PDA and C–O groups, or simply because the layer formed over the CNC surface reduces the contribution of C–O groups in the spectrum. Thus, the decrease in intensity or disappearance of the peak at 1110 cm^−1^ in CNC@PDA can be interpreted as a masking or partial blocking effect of the C–O vibrations due to the PDA layer on the surface of the CNC.

The peak at 1015 cm^−1^ indicates the presence of ether bonds (C–O–C) in the CNC structure, reflecting the quantity and organization of these bonds in the sample [27]. In PDA, this peak may appear with lower intensity or be less pronounced, as PDA does not have the pyranose rings found in CNC. After the modification of CNC, the peak at 1015 cm^−1^ may exhibit reduced intensity or an altered shape compared to the peak in unmodified CNC. This occurs because PDA can interfere with the structure of CNC, altering the organization and intensity of the C–O–C vibrations.

### 3.4. Thermogravimetric Analysis (TGA)

**Figure 4:**
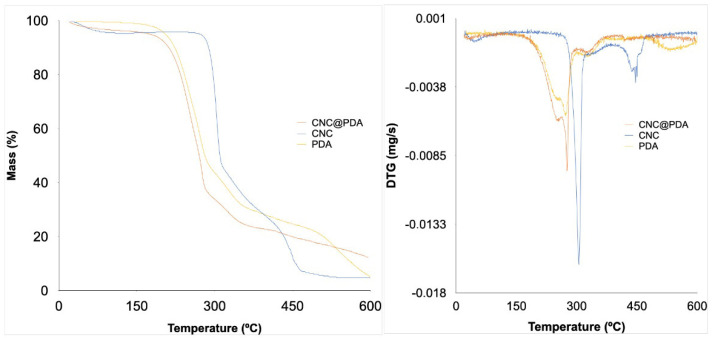
Thermogravimetric (TG) and derivative thermogravimetric (DTG) curves.

In Figure 4, the thermal behavior of PDA and both the original and modified CNC was evaluated.

The thermal analysis in Figure 4 shows that the modification of CNC with PDA significantly affects their thermal behavior. Initially, a weight loss of the materials (CNC and CNC@PDA) is observed below 100 °C, attributed to moisture evaporation. The thermal degradation of CNC typically occurs in two main stages. The first stage, starting around 240 °C, is associated with the depolymerization of the cellulose chain, meaning the cleavage of glycosidic (β-1,4) bonds, releasing smaller fragments. The second stage, occurring at approximately 350 °C, involves the decomposition of smaller molecules resulting from depolymerization, leading to the formation of carbonaceous residues, in line with previous studies [28,29]. This degradation is generally influenced by the crystallinity of CNC: higher crystallinity tends to delay thermal decomposition due to the compact and ordered structure that offers resistance to thermal degradation. The final carbon residue of CNC was 4.44%.

On the other hand, PDA can reduce the thermal stability of the CNC@PDA, initiating decomposition at lower temperatures (around 170 °C). This can be attributed to several factors: (i) PDA has a complex and heterogeneous chemical structure, containing various functional groups, such as catechols, amines and quinones, which may begin to decompose at lower temperatures due to their higher chemical reactivity. PDA is formed by the polymerization of dopamine, and its amorphous structure may lead to easier decomposition compared to the crystalline structure of CNC [23,30]. (ii) The modification of CNC with PDA may introduce new chemical interactions at the CNC@PDA interface, such as hydrogen bonds and π-π interactions, which affect thermal stability. The lower thermal stability of the PDA layer compared to pure CNC is attributed to the degradation of its functional groups, which occurs at lower temperatures [31,32]. (iii) The presence of PDA on the CNC surface can create a different thermal barrier, resulting in uneven heat distribution and, consequently, less uniform thermal decomposition [31,32,33].

A modification of CNC with PDA alters the thermal stability of the material due to the formation of both physical and chemical interactions between PDA and the CNC surface. Catechol groups in PDA can react with oxygen at lower temperatures, while PDA adsorption affects crystallization characteristics and influences thermal decomposition, contributing to premature degradation. These modifications arise from physical interactions rather than necessarily involving strong new chemical bonds, affecting the overall thermal behavior of the resulting composite. Previous studies indicate that surface modification of CNC with different agents, including polymers, generally results in reduced thermal stability due to the addition of components that begin to degrade at lower temperatures [34]. However, the modification with PDA in this study indicates a thermal behavior that could suggest a decrease in the initial decomposition temperature, although this specific behavior requires more detailed investigation for definitive confirmation.

### 3.5. Investigation of the Antimicrobial Activity of PDA Solution and CNC@PDA Suspensions

**Figure 5:**
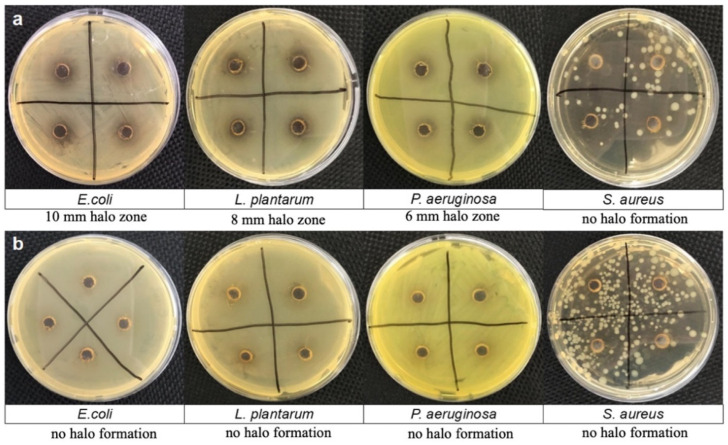
Photographs of bacterial inhibition halos on agar plates: (**a**) PDA solution and (**b**) CNC@PDA suspension.

The antimicrobial activity of PDA solution and CNC@PDA suspensions was determined using a standard well diffusion method against gram-negative bacteria (*Escherichia coli* ATCC 11229 and *Pseudomonas aeruginosa* ATCC 6538) and gram-positive bacteria (*Staphylococcus aureus* ATCC 15442 and *Lactobacillus plantarum* ATCC 8014). The qualitative antimicrobial activity was assessed by monitoring the ability of each material to inhibit bacterial growth. The bacterial inhibition zones were observed on agar plates and are shown in Figure 5a for the PDA solution and Figure 5b for the CNC@PDA suspension.

The PDA solution demonstrated antimicrobial properties, evidenced by clear inhibition zones around the wells on agar plates (Figure 5a). An increase in zone diameters was observed with the addition of PDA: 10 mm for *E. coli*, 8 mm for *L. plantarum* and 6 mm for *P. aeruginosa*. The *S. aureus* bacteria growth was heterogeneous, disturbing halo formation. However, reduction in the bacteria growth can be observed when PDA pristine was evaluated compared to CNC@PDA compound.

The difficulty in exposing the functional groups of PDA arises from its adsorption on the CNC surface. This adsorption prevents active groups, such as catechols and amines, from freely interacting with the external environment. As a result, the reduced availability of these groups for bacterial interaction decreases the antimicrobial activity of CNC@PDA. Furthermore, the lower number of *S. aureus* observed on the agar plate, despite consistent bacterial suspension concentrations, can be attributed to its specific interaction dynamics with PDA. As a gram-positive bacterium, *S. aureus* has a thicker peptidoglycan layer, which may reduce susceptibility to the reactive oxygen species (ROS) generated by PDA compared to gram-negative bacteria. Additionally, the lack of an outer membrane in gram-positive bacteria could diminish the effects of ROS damage on *S. aureus*.

The evidence suggests that PDA possesses antimicrobial properties, possibly due to the generation of reactive oxygen species (ROS). ROS are formed when oxygen molecules (O_2_) are reduced, partially or completely, through electron transfer. In PDA, dopamine polymerization involves oxidation and reduction processes that create different redox states. PDA can transfer electrons to molecular oxygen, generating ROS such as hydrogen peroxide (H_2_O_2_), superoxide anion (O_2_^−^) and hydroxyl radical (OH^−^). These reactive species can damage bacterial structures, contributing to the antimicrobial activity of PDA.

**Figure 6:**
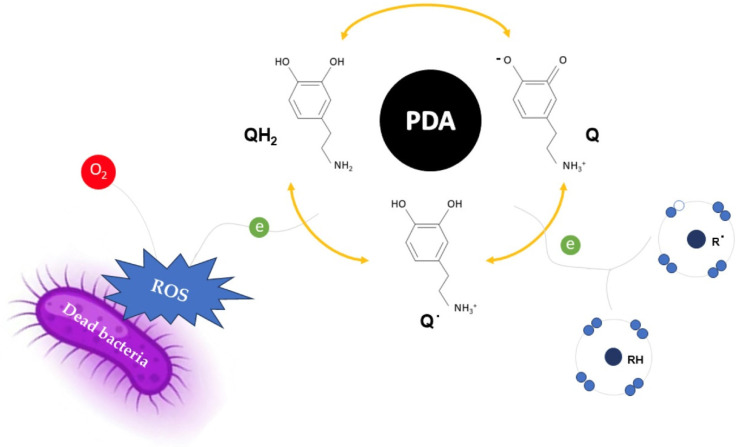
The redox activity of PDA provides either a beneficial antioxidant effect by extinguishing oxidative free radicals or, alternatively, a harmful pro-oxidant effect by donating electrons to O_2_, generating reactive oxygen species (ROS) that confer antimicrobial properties to PDA (QH_2_, Q and Q˙ represent the catechol, quinone and semiquinone groups of PDA, respectively).

Due to the dynamic process dependent on environmental conditions (pH, temperature, oxygen), polymerization occurs as long as dopamine is present under the appropriate conditions. Electron transfer allows the coexistence of three species: reduced catechol (QH_2_), semiquinone radical (Q˙) and oxidized o-quinone (Q). PDA can accept electrons from reducing agents and donate them to various oxidants. Depending on the conditions, electron donation can result in either a beneficial antioxidant effect, neutralizing free radicals or a pro-oxidant effect, generating ROS by donating electrons to O_2_ (Figure 6) [22].

The study by Liu et al. (2019) investigated the antimicrobial activities of PDA nanoparticles and revealed that ROS are potent antimicrobial agents against various bacteria, including *E. coli*, *P. aeruginosa*, *S. aureus* and methicillin-resistant *S. aureus* (MRSA) [22]. In contrast, the study by Shumbula et al. (2022) showed that pure PDA exhibited no antimicrobial activity against *E. coli* and *S. aureus* strains, as no clear inhibition zones were observed [30]. The results indicate that the antimicrobial efficacy of PDA depends on its redox state, likely attributed to its ability to generate ROS. In this study, which explores the characterization of CNC and PDA, these findings are corroborated, highlighting the importance of ROS generation for PDA’s antimicrobial activity.

### 3.6. Mechanical Properties

**Figure 7:**
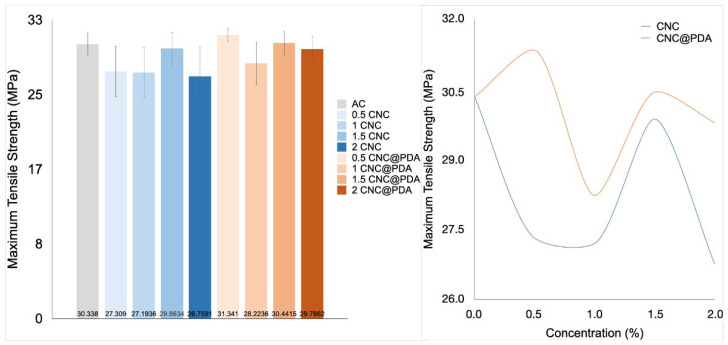
Maximum tensile strength of films: on the left side media and standard deviation of CA film pristine and incorporated with CNC (0, 0.5, 1.0, 1.5, and 2.0%) and CNC@PDA (0, 0.5, 1.0, 1.5, and 2.0%) and on the right side the variation under the concentration of CNC and CNC@PDA.

**Figure 8:**
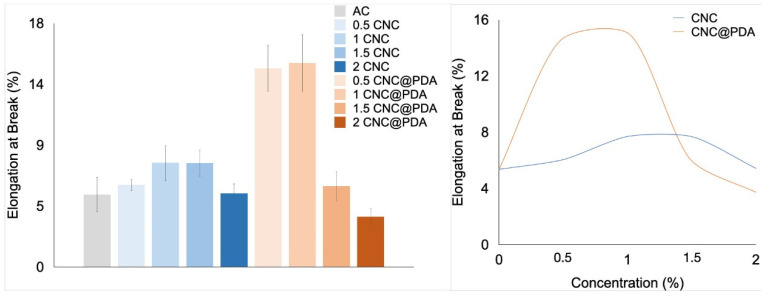
Elongation at break of films: on the left side media and standard deviation of CA film pristine and incorporated with CNC (0, 0.5, 1.0, 1.5, and 2.0%) and CNC@PDA (0, 0.5, 1.0, 1.5, and 2.0%) and on the right side the variation under the concentration of CNC and CNC@PDA.

To study the mechanical properties of CA films incorporated with CNC and CNC@PDA, tensile (Figure 7) and flexibility tests (Figure 8) were performed on the films.

The statistical difference in tensile strength between samples with 2% CNC and 0.5% CNC@PDA can be explained by various factors related to the interaction between CNC@PDA and the CA matrix. The 0.5% CNC@PDA sample may have reached an optimal concentration, favoring the formation of effective networks within the polymer matrix, which enhances tensile strength. In contrast, the 2% CNC sample may have an excessive concentration, resulting in aggregation or uneven distribution of CNC, weakening the material.

The efficiency of CNC dispersion within the polymer matrix tends to vary with concentration. At lower concentrations, such as 0.5%, CNCs are more likely to be well dispersed and interact effectively with the matrix, improving mechanical properties. On the other hand, at higher concentrations such as 2%, aggregation may occur, leading to defects and reduced tensile strength.

Additionally, the presence of PDA can aid in the formation of networks that enhance stress transfer and dispersion. However, if the CNC@PDA concentration is too high, an excessive network may form, offering diminishing returns or even negatively impacting mechanical properties. The compatibility between the modified CNC and the matrix can also vary with concentration; more effective interaction occurs at specific concentrations, resulting in better mechanical performance, while at other concentrations, this interaction may be less efficient [35,36].

The 2% CNC concentration resulted in an excessively rigid network, compromising flexibility. The low compatibility between the polar CNC charges and the non-polar matrix may lead to increased defect formation and porosity, weakening the material’s mechanical properties. These defects act as weak points that reduce the material’s ability to deform, negatively impacting its flexibility [37].

In contrast, 0.5% and 1% CNC@PDA concentrations improved phase compatibility and promoted a more flexible structure. PDA enhanced the interaction between components, promoted more homogeneous dispersion and prevented rigid agglomeration, essential for greater flexibility [12,38]. Moreover, the presence of PDA may have filled pores and defects in the matrix, preventing weak points that would impair the material’s ability to deform. However, higher concentrations (1.5% and 2% CNC@PDA) made the matrix excessively rigid and less flexible. PDA may have generated excessive interactions between polymer chains, resulting in a dense and rigid structure. This rigidity hinders molecular mobility and the material’s ability to deform, compromising its flexibility [38].

Despite the observed trends, the ANOVA analysis did not indicate statistical significance. This means that the differences between the analyzed conditions cannot be considered conclusive from a statistical point of view. However, these trends still present potential relevance for understanding the system, offering important insights based on experimental observations and theoretical support, which can serve as a foundation for future investigations.

## 4. Conclusions

Experimental and theoretical analyses indicate the surface modification of cellulose nanocrystals. The results indicate that the antimicrobial activities of PDA are redox-dependent and are presumably conferred by its ROS-generating abilities.

The zeta potential and polydispersity index (PDI) showed that PDA modification improves the colloidal stability of CNC in solution. Additionally, PDA acts as a bridge to enhance interfacial adhesion between CNC and the hydrophobic matrix, thereby improving the mechanical properties of the composite.

Mechanical tests revealed that the addition of PDA affects the tensile strength of the films. The 1% CNC@PDA concentration demonstrated the best balance between tensile strength and flexibility, suggesting that this concentration provides an optimal network within the matrix, improving stress distribution and allowing greater deformation without fractures. Thus, the modification of CNC with PDA provided significant benefits for the colloidal stability and mechanical properties of the cellulose acetate films.

## Figures and Tables

**Table 1:** Hydrodynamic diameter, polydispersity index and ζ potential.

Treatment *	Hydrodynamic Diameter (nm)	Polydispersity Index	Zeta Potential (mV)
CNC	86.0 ± 2.19 ^a^	0.3 ± 0.01 ^a^	−67.5 ± 0.87 ^b^
PDA	273.9 ± 23.06 ^b^	0.6 ± 0.19 ^b^	−44.0 ± 2.55 ^a^
CNC@PDA	274.9 ± 3.27 ^b^	08 ± 0.04 ^c^	−39.0 ± 1.36 ^a^

^a,b,c^ Different letter superscripts in the same column indicate a statistically significant difference (*p* < 0.05). * CNC: cellulose nanocrystals; PDA: polydopamine; CNC@PDA: cellulose nanocrystals modified with polydopamine.

## Data Availability

The original contributions presented in this study are included in the article. Further inquiries can be directed to the corresponding author.

## Abbreviations

CNCs	Cellulose nanocrystals
PDA	Polydopamine
CNC@PDA	PDA-coated CNC
ROS	Reactive oxygen species
AC	Cellulose acetate
PVA	Polyvinyl alcohol
PEG	Polyethylene glycol
SEM	Scanning electron microscopy
FTIR	Fourier-transformed infrared absorption spectroscopy
TGA	Thermogravimetric analysis
PDI	Polydispersity index
DLS	Dynamic light scattering
ATR	Attenuated reflectance
